# Pancreatitis as a Pulmonary Pathology: A Rare Case of a Pancreaticopleural Fistula Presenting as Recurrent Pleural Effusions Causing Mediastinal Shift

**DOI:** 10.7759/cureus.64246

**Published:** 2024-07-10

**Authors:** Zidan Saleh, Resham Pawar, Ashwin Pillai, Ahmed Abdelwahed, Omar Ibrahim

**Affiliations:** 1 Internal Medicine, University of Connecticut Health, Farmington, USA; 2 Pulmonary, Critical Care, and Sleep Medicine, University of Connecticut Health, Farmington, USA

**Keywords:** cystogastrostomy, endoscopic ultrasound (eus), mediastinal shift, pleural effusion, pancreaticopleural fistula, pseudocyst, pancreatitis, case report

## Abstract

Pancreaticopleural fistula (PPF) is a rare complication of chronic pancreatitis and pancreatic pseudocyst. It can present as recurrent pleural effusions and can be difficult to diagnose and treat. We present the case of a 37-year-old male with a history of chronic idiopathic pancreatitis complicated by a pseudocyst who came in with progressive dyspnea, cough, and pleuritic chest pain. The chest X-ray on presentation showed near-complete opacification of the left hemithorax, suggesting a large pleural effusion. Upon thoracentesis, black-bloody fluid was drained, and the pleural fluid analysis was consistent with an exudate with significantly elevated levels of amylase, lipase, and bilirubin. Cytology revealed abundant lipofuscin-laden macrophages, suggesting an intra-abdominal source of the accumulated fluid. A post-drainage CT of the chest showed the resolution of the pleural effusion and an interval decrease in the pancreatic pseudocyst size, indicating a fistulous connection to the pleural space. An endoscopic ultrasound (EUS) was performed with efforts to perform cystogastrostomy aspiration that was hindered by the interference of splenic vasculature obstructing the needle’s path. The patient was transferred to another facility for definitive treatment with surgical pancreatectomy and auto islet cell transplant. This case underscores the importance of considering PPF as a possible diagnosis, especially in cases of recurrent pleural effusions and a history of pancreatitis and pancreatic pseudocyst. It also emphasizes the significance of EUS as the preferred modality for pseudocyst evaluation and its potential for minimally invasive treatment.

## Introduction

Pancreatic inflammatory conditions resulting in acinar and ductal cell dysfunction can lead to severe outcomes [1]. Pleural effusions caused by pancreaticopleural fistulas (PPFs) are exceedingly rare, occurring in around 0.4% of chronic pancreatitis patients and accounting for 1% of pleural effusion cases [2,3]. PPFs are primarily caused by a pancreatic pseudocyst or disruption of the main pancreatic duct. They are most commonly observed in individuals who have consumed alcohol and, more frequently, in men [4,5]. Inflammation caused by pancreatic digestion enzymes results in the formation of a path from the pancreas to the retroperitoneum, resulting in the collection of fluid and the possible development of a pseudocyst and eventual communication with the pleural cavity [6]. Pleural effusions caused by PPFs are recurring and usually positive for amylase and lipase [4]. Thoracentesis typically reveals exudative straw-colored or serosanguinous pleural fluid [7,8]. Over 75% of instances occur on the left side. We present this case to raise awareness of PPF as a potential cause of significant pleural effusion and highlight the non-invasive and invasive treatment options to prevent its recurrence.

## Case presentation

A 37-year-old male with a history of chronic idiopathic pancreatitis complicated by pseudocyst formation, calcifications, and pancreatic duct dilatation presented at the emergency department with shortness of breath that had worsened during the previous two weeks. It was associated with a dry cough and left-sided pleuritic chest pain. He denied having a fever, chills, hemoptysis, vomiting, abdominal discomfort, or diarrhea and claimed to have no recent travel history or interaction with sick people. The patient had had three episodes of severe pancreatitis in the previous year and was diagnosed with pancreatic pseudocyst two years before this admission. He was admitted a month before his current presentation for a pleural effusion and thoracentesis. His medications included pancreatic enzymes and rivaroxaban for the treatment of portal vein thrombosis two years ago, which he stopped taking after three months due to non-compliance.

When the patient arrived at the emergency department, his vital signs were as follows: 36.1°C, blood pressure of 153/107 mmHg, heart rate of 86 beats/minute, respiration rate of 26 breaths/min, and oxygen saturation of 97% on room air.

During the physical examination, the patient seemed uncomfortable and in considerable distress. Breath sounds were absent on the left side of the chest, with dullness to percussion. Rightward tracheal deviation was observed, with no jugular venous distention. Abdominal and other examinations showed no remarkable findings.

**Figure 1:**
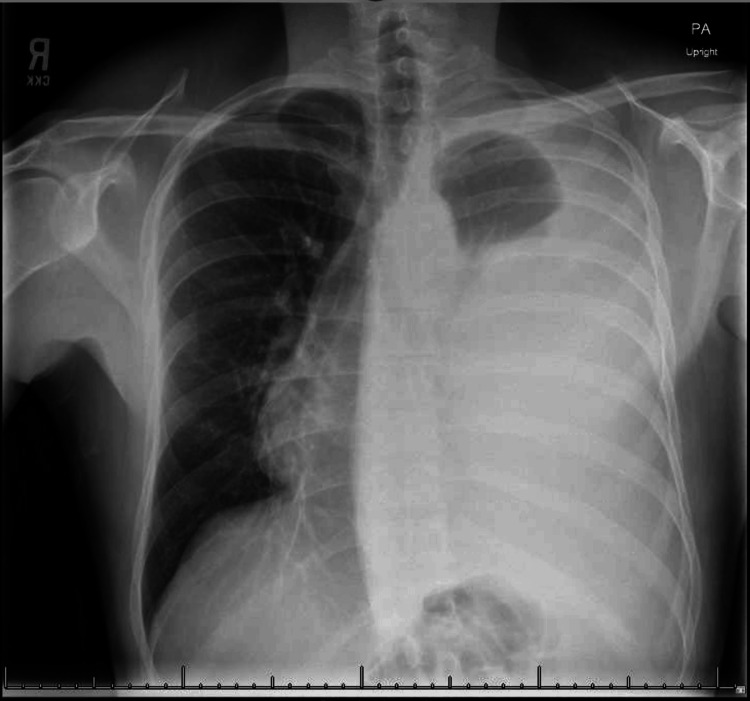
Chest radiograph demonstrating a left-sided massive pleural effusion causing near-total opacification of the left hemithorax and a rightward mediastinal shift.

The initial chest radiograph revealed almost total opacification on the left side of the chest, as well as rightward tracheal deviation (Figure 1). The lab results showed a white blood cell count of 7.9 × 10^9^/L, a hemoglobin level of 12.3 g/L, and a platelet count of 365,000 platelets/μL. The patient’s serum creatinine level was 0.90 g/dL, and the liver function panel findings were within the normal range.

The patient underwent a left thoracentesis, and a chest tube was inserted, which resulted in the prompt removal of 2.5 L of black-bloody fluid. The pleural fluid analysis revealed 762 nucleated cells/mm^3^, with 6% polymorphonuclear leukocytes, 86% monocytes/macrophages, 6% lymphocytes, and 2% eosinophils. The fluid glucose levels were 96 mg/dL, lactate dehydrogenase (LDH) was 1,176 U/L, and fluid protein level was 4.7 g/dL compared to the serum glucose level of 134 mg/dL, total protein level of 6.6 g/dL, and LDH level of 261 U/L. These findings were compatible with an exudative pleural effusion. In addition, further pleural fluid testing revealed increased levels of amylase (21,293 U/L), lipase (57,666 U/L), and bilirubin (7.5 mg/dL). Pleural fluid triglycerides and cholesterol were normal at 66 mg/dL (target range: <110 mg/dL) and 42 mg/dL (reference range: <45 mg/dL), respectively. Fluid cytology, culture, and Gram stain were all negative. Finally, the cytology revealed no malignancy but an abundance of lipofuscin-laden macrophages.

**Figure 2:**
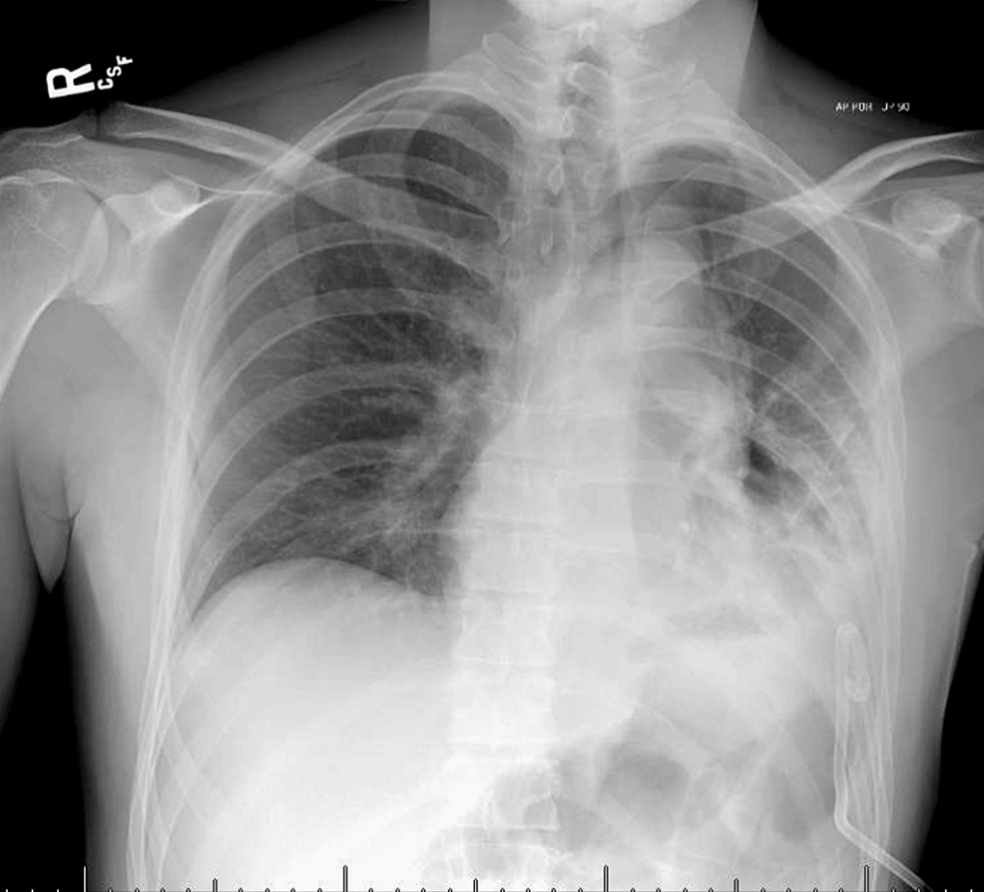
Post-chest tube placement chest radiograph demonstrating interval decrease in the left pleural effusion with a small left-sided pneumothorax.

A post-thoracentesis chest radiograph demonstrated a significant interval decrease in the pleural effusion with a resolution of the mediastinal shift, improved aeration in the left lung field, and interval development of a small pneumothorax. A total of 5,500 mL was drained through the chest tube during the admission (Figure 2).

**Figure 3:**
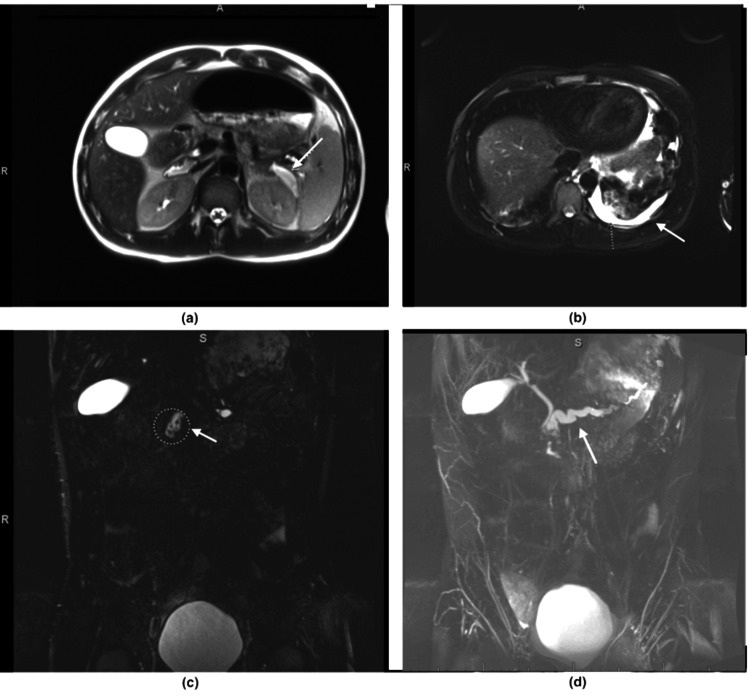
Magnetic resonance cholangiopancreatography demonstrating the pancreatic pseudocyst. Arrow in (a) and (c) along with left-sided pleural effusion (b), and a dilated pancreatic duct indicated by the arrow in (d).

Magnetic resonance cholangiopancreatography (MRCP) showed significant dilatation of the pancreatic duct up to 1.1 cm, with multiple stones present in the main pancreatic duct. There was also a retroperitoneal pseudocyst in the left upper quadrant that connected with the main pancreatic duct, measuring 4.6 × 1.4 × 3.5 cm (Figure 3).

**Figure 4:**
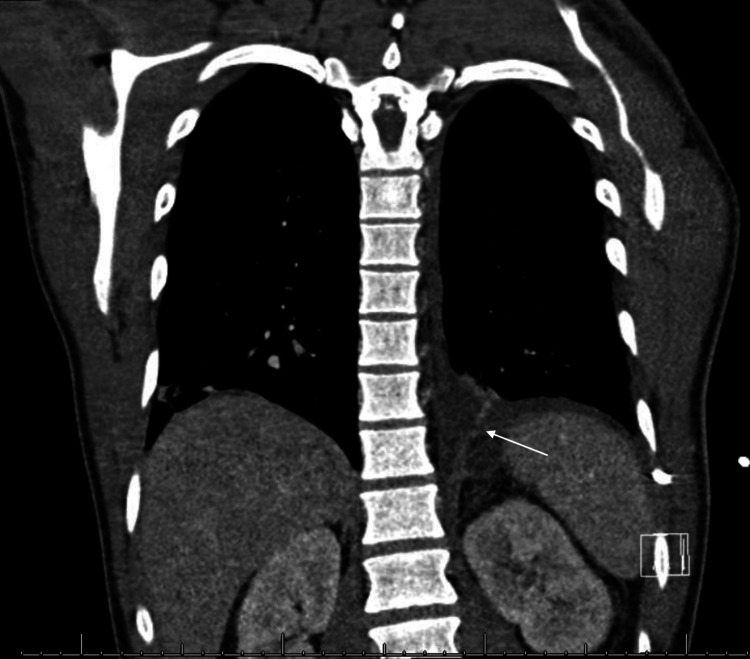
Post-thoracentesis chest CT with intravenous contrast demonstrating a probable communicating fistula between the sub-diaphragmatic and left pleural spaces (white arrow).

After the thoracentesis procedure, a CT scan of the chest with intravenous contrast was performed to investigate the cause of the pleural effusion. The scan revealed a resolution of the pleural effusion and reduction of the pancreatic pseudocyst size, with a suspicious fistulous connection to the pleural space. (Figure 4).

**Figure 5:**
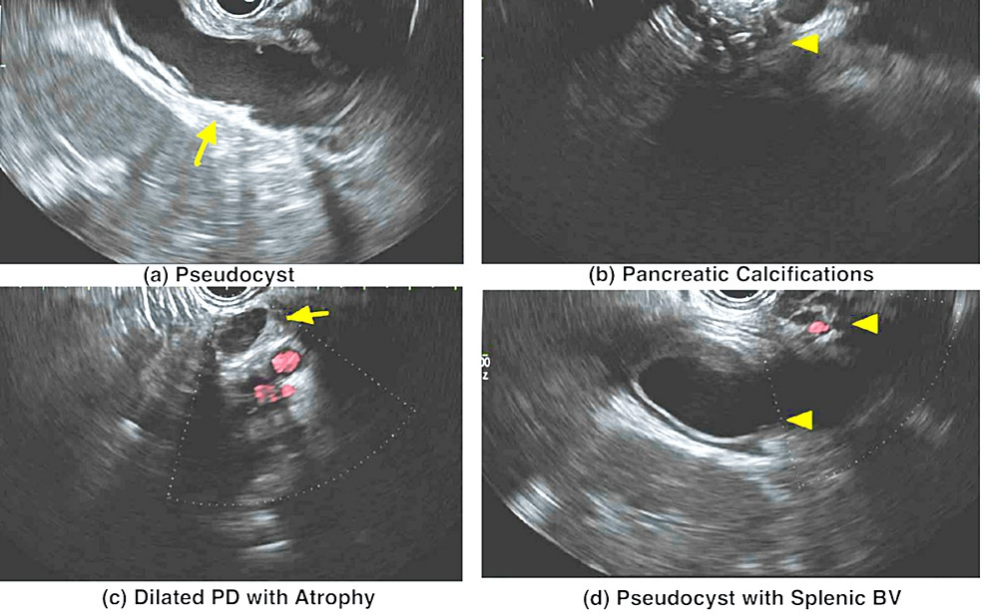
EUS demonstrating (a) a pseudocyst (yellow arrow), (b) pancreatic calcifications (arrowhead), (c) dilated PD with atrophy, and (d) the splenic blood vessels close to the pseudocyst. EUS: endoscopic ultrasound; PD: pancreatic duct; BV: blood vessels

During the esophagogastroduodenoscopy with endoscopic ultrasound (EUS), a relatively normal upper endoscopy was observed. The EUS revealed a normal-appearing biliary system and chronic calcific pancreatitis with a significant stone burden. Pancreatic duct dilatation with parenchymal atrophy was noted. Additionally, a thick-walled (anechoic to hypoechoic) cystic lesion was found at the tail of the pancreas, consistent with a pseudocyst. Unfortunately, cystogastrostomy aspiration could not be performed due to the intervening splenic vasculature between the transducer and the pseudocyst (Figure 5).

Upon multidisciplinary team discussion, it was decided to transfer the patient to another facility for evaluation for definitive therapy, including surgical intervention with pancreatectomy, auto islet cell transplant, and fistulectomy.

## Discussion

PPF is an uncommon complication of acute or chronic pancreatitis, with an incidence of 0.4% [2]. PPF is most commonly related to alcohol pancreatitis, but it can also be associated with trauma or iatrogenic injury [4]. The pathophysiology of PPF involves the formation of a posterior pathway from the pancreatic duct to the pleura or, more commonly, communication between an incompletely formed or ruptured pseudocyst, usually through the aortic or esophageal hiatus and finally penetrating through the pleural cavity [9]. CT imaging and MRCP are commonly used to diagnose anomalies in the pancreatic duct and parenchyma. Endoscopy using EUS and possibly ERCP is critical in the diagnosis and possible management of pancreatic duct dilatation, stone burden, and the detection of pseudocysts, all of which were investigated in this case. Given the quick recurrence of the pleural effusion within a month, we proposed a PPF in our patient. However, CT, EUS, and MRCP tests revealed no evidence of this condition. Furthermore, the size of the pancreatic pseudocyst decreased over time compared to a previous CT, indicating a probable rupture.

Most patients with PPFs are treated conservatively with thoracentesis, parenteral nutrition, and octreotide to reduce pancreatic secretions, which work in only 40-60% of cases [10-12]. If the fistula does not resolve, as in this case with recurrent pleural effusions, an alternative treatment, such as surgery or minimally invasive procedures, should be considered because mortality may increase otherwise [13].

EUS is the most recommended technique for evaluating pancreatic pseudocysts because it can measure the distance between the gastrointestinal tract lumen and the pseudocyst and the presence of vascular components [14]. Furthermore, it can be more useful when a fine-needle aspiration is performed, followed by cytology and tumor marker analysis. It can also be used for minimally invasive surgeries to evacuate the pseudocyst, such as cystogastrostostomy, cystoduodenostomy, or cystojejunostomy, depending on the location of the pseudocyst. In this case, EUS was employed to aid in the diagnosis. However, cystogastrostomy could not be performed because the splenic vasculature obstructed the needle’s passage.

Surgical excision of the pseudocyst is recommended in cases of painful chronic pancreatitis, the presence of multiple pseudocysts, gastrointestinal bleeding caused by a pseudoaneurysm, common bile duct obstruction, cyst neoplasia, involvement of the splenic vein, and technical challenges in draining the pseudocyst, as demonstrated in our case [15].

## Conclusions

PPF is challenging to diagnose and treat. Clinicians should be suspicious, especially if there are recurrent left-sided pleural effusions and a history of acute or chronic pancreatitis. Diagnostic and therapeutic thoracentesis should be considered early, as pleural fluid amylase can aid in diagnosis and prevent treatment delays. EUS is the preferred method for evaluating pseudocysts and provides a therapeutic option through minimally invasive treatments such as cystogastrostomy drainage. Surgical excision should be considered in recurrent pleural effusions as it has been shown to reduce morbidity and mortality.
